# Spontaneous hepatic tumor regression in an infant with trisomy 18

**DOI:** 10.1002/ped4.70063

**Published:** 2026-04-29

**Authors:** Masashi Hotta, Katsuya Hirata, Shota Inoue, Kohei Higuchi, Eriko Nishi, Kazuko Wada

**Affiliations:** ^1^ Department of Neonatal Medicine Osaka Women's and Children's Hospital Izumi Japan; ^2^ Laboratory of Vaccine Materials/Laboratory of Gut Environmental System, Microbial Research Center for Health and Medicine, National Institutes of Biomedical Innovation, Health and Nutrition Ibaraki Japan; ^3^ Department of Hematology/Oncology Osaka Women's and Children's Hospital Izumi Japan; ^4^ Department of Medical Genetics Osaka Women's and Children's Hospital Izumi Japan

To the Editor:

Hepatic tumors are relatively common in infants with trisomy 18 syndrome (T18).^1^ Although recent studies report successful management of hepatoblastoma in such patients,^2^, ^3^ multiple comorbidities often cause clinical instability, limiting treatment options. We report the case of an infant with T18 whose hepatic tumor regressed spontaneously after oncologic treatment was deferred due to poor clinical status.

A female infant with T18, complicated by a ventricular septal defect and progressive pulmonary hypertension, was born at 37 weeks of gestation. After neonatal intensive care, the patient was discharged at 5 months of age, with home respiratory support via high‐flow nasal cannula. At 1 year and 3 months of age, the serum alpha‐fetoprotein (AFP) level was elevated to 402.4 ng/mL (normal <87 ng/mL) (Figure 1A). Magnetic resonance imaging showed a 17 × 23 × 26 mm mass in Couinaud segment 5 (S5) and a 5 mm mass in S7 (Figure 1B), both strongly suggestive of hepatoblastoma on contrast‐enhanced imaging (Figure 1C). Chemotherapy was planned; however, treatment was deferred due to infections and clinical instability.

**FIGURE 1:**
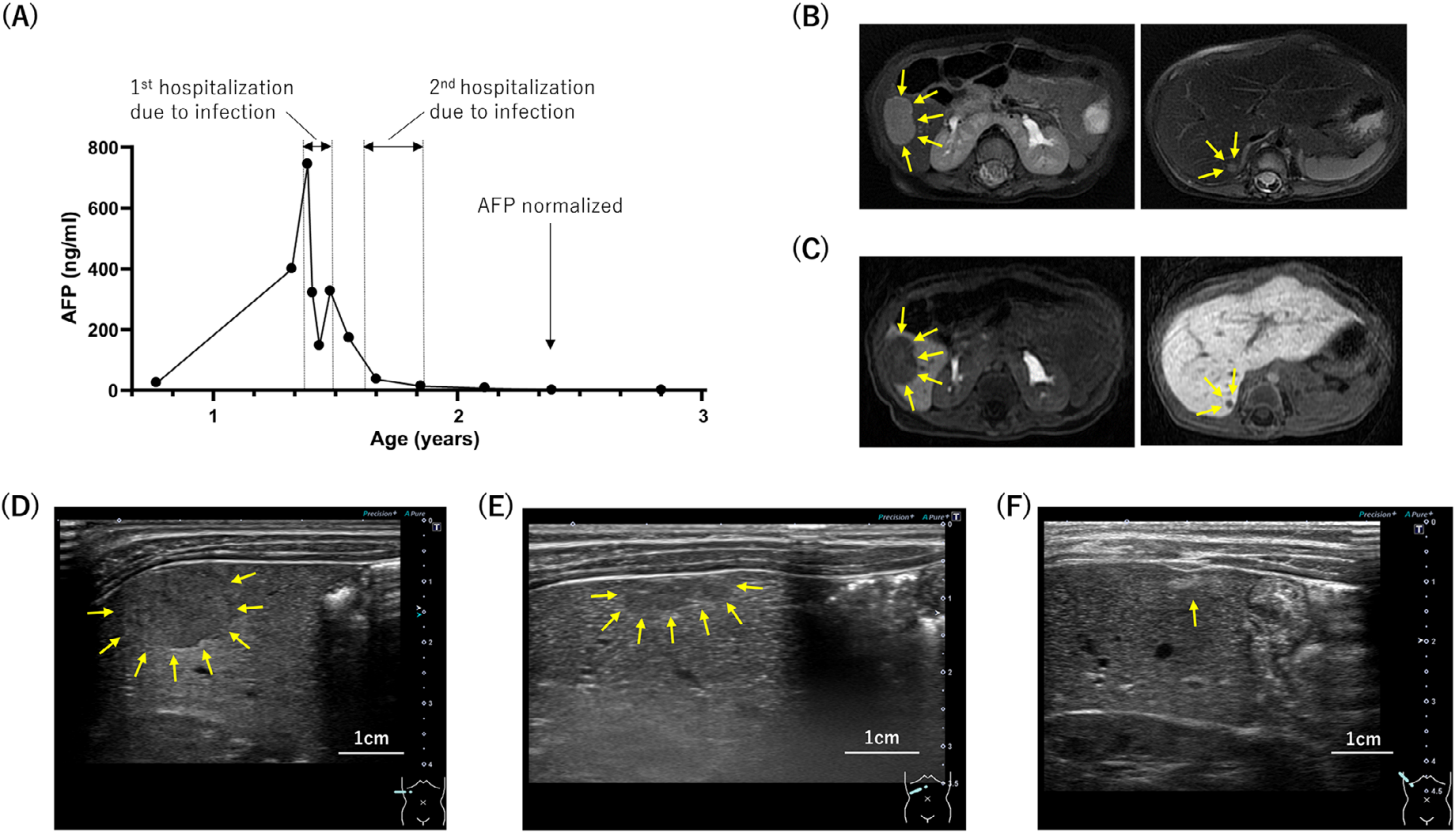
(A) Changes in serum alpha‐fetoprotein (AFP) levels. (B) T2‐weighted magnetic resonance and (C) hepatobiliary phase images with gadolinium‐ethoxybenzyl‐diethylenetriaminepentaacetic acid enhancement displaying tumors located in Couinaud segments 5 (left) and 7 (right) at 1 year and 2 months of age. (D–F) Hepatic ultrasonography of the tumor in the Couinaud segment 5 at 1 year and 5 months, 1 year and 10 months, and 2 years and 9 months of age, respectively. The tumor gradually decreased, with only a residual scar at 2 years and 9 months of age.

The tumors regressed spontaneously during two subsequent hospitalizations for infections requiring intensive care. The patient received cefotaxime, ampicillin, nitric oxide, macitentan, dobutamine, fentanyl, dexmedetomidine, midazolam, and diazepam for worsening pulmonary hypertension and supportive care. The AFP level, which peaked at 747.5 ng/mL, declined to 17.3 ng/mL by 1 year and 7 months of age (Figure 1A) without chemotherapy. Follow‐up imaging revealed marked reduction, with residual scarring and no visible tumors (Figure 1D–F). The patient remained free of recurrence through 6 years of age.

To our knowledge, this is the first pediatric report of spontaneous regression of primary hepatic tumors in a child with T18. The mechanisms underlying this regression remain unclear. Potential contributors include tumor hypoxia secondary to pulmonary hypertension and infection‐related systemic inflammatory responses that may enhance immune‐mediated tumor suppression.^4^

Although surgery and chemotherapy remain treatment options for hepatic tumors, this case suggests that deferring such therapy may be appropriate in clinically unstable patients. Continued use of condition‐tailored approaches based on the best available evidence remains crucial.^5^ Comprehensive management, regular monitoring, and clear communication with families are also essential. Such care supports alignment with the patient's best interests and highlights the importance of sustained medical support and family‐centered care throughout the clinical course.

## CONFLICT OF INTEREST

The authors declare no conflict of interest.

## CONSENT FOR PUBLICATION

Written informed consent was obtained from the infant's parents. Personal information has been anonymized to ensure privacy and confidentiality.

## References

[1] Farmakis SG , Barnes AM , Carey JC , Braddock SR . Solid tumor screening recommendations in trisomy 18. Am J Med Genet A. 2019;179:455–466. DOI: 10.1002/ajmg.a.61029 30637956

[2] Lucas DJ , Rubinstein J , Gosain A , Tiao G, Head T, Pratap JN, et al. Surgical and anesthetic management for hepatectomy in two pediatric patients with trisomy 18, pulmonary hypertension, and hepatoblastoma. Pediatr Blood Cancer. 2019;66:e27678. DOI: 10.1002/pbc.27678 30803146

[3] Inoue A , Suzuki R , Urabe K , Kawamura Y, Masuda M, Kishi K, et al. Therapeutic experience with hepatoblastoma associated with trisomy 18. Pediatr Blood Cancer. 2018;65:e27093. DOI: 10.1002/pbc.27093 29701292

[4] Sakamaki A , Kamimura K , Abe S , Tsuchiya A, Takamura M, Kawai H, et al. Spontaneous regression of hepatocellular carcinoma: a mini‐review. World J Gastroenterol. 2017;23:3797–3804. DOI: 10.3748/wjg.v23.i21.3797 28638219 PMC5467065

[5] Pyle AK , George TN , Cummings JJ , Laventhal NT; Committee on Bioethics , Section on Neonatal‐Perinatal Medicine , et al. Guidance for caring for infants and children with trisomy 13 and trisomy 18: clinical report. Pediatrics. 2025;156:e2025072719. DOI: 10.1542/peds.2025-072719 40685149

